# Corrigendum

**DOI:** 10.1093/gbe/evab249

**Published:** 2021-12-13

In the originally published version of the below papers, there was an error in the Data Availability statement.

Universal Constraints on Protein Evolution in the Long-Term Evolution Experiment with *Escherichia coli*

*Genome Biology and Evolution*, Volume 13, Issue 6, June 2021, evab070, https://doi.org/10.1093/gbe/evab070

Selection Maintains Protein Interactome Resilience in the Long-Term Evolution Experiment with *Escherichia coli*

*Genome Biology and Evolution*, Volume 13, Issue 6, June 2021, evab074, https://doi.org/10.1093/gbe/evab074

The incorrect sentence was: “The data and analysis codes underlying this article are available on the Dryad Digital Repository (doi: 10.5061/dryad.x0k6djhj5).”

The correct sentence is: “The data and analysis codes underlying this article are available on the Zenodo Digital Repository (https://doi.org/10.5281/zenodo.5585210).”

These errors have been corrected online.

